# Pattern Synthesis of Linear Antenna Array Using Improved Differential Evolution Algorithm with SPS Framework

**DOI:** 10.3390/s20185158

**Published:** 2020-09-10

**Authors:** Ruimeng Zhang, Yan Zhang, Jinping Sun, Qing Li

**Affiliations:** 1 School of Electronics & Information Engineering, Beihang University, Beijing 100191, China; zhangruimeng@buaa.edu.cn (R.Z.); yanzhang@buaa.edu.cn (Y.Z.); 2 Department of Engineering, University of Cambridge, Cambridge CB12PZ, UK; ql289@cam.ac.uk

**Keywords:** random optimization algorithm, differential evolution algorithm, SPS framework, SPS-JADE algorithm, antenna array, sidelobe suppression, null depth, pattern synthesis

## Abstract

In this paper, an improved differential evolution (DE) algorithm with the successful-parent-selecting (SPS) framework, named SPS-JADE, is applied to the pattern synthesis of linear antenna arrays. Here, the pattern synthesis of the linear antenna arrays is viewed as an optimization problem with excitation amplitudes being the optimization variables and attaining sidelobe suppression and null depth being the optimization objectives. For this optimization problem, an improved DE algorithm named JADE is introduced, and the SPS framework is used to solve the stagnation problem of the DE algorithm, which further improves the DE algorithm’s performance. Finally, the combined SPS-JADE algorithm is verified in simulation experiments of the pattern synthesis of an antenna array, and the results are compared with those obtained by other state-of-the-art random optimization algorithms. The results demonstrate that the proposed SPS-JADE algorithm is superior to other algorithms in the pattern synthesis performance with a lower sidelobe level and a more satisfactory null depth under the constraint of beamwidth requirement.

## 1. Introduction

An array of sensors or antennas, compared with a single one, often displays better characteristics and can perform more functions. One example is the structural health monitoring (SHM) system based on the finite element theory [1], which utilizes a sensor array. This paper focuses on the antenna arrays which have the merits of high gains, flexible scanning, and easy beamforming implementations, and which have therefore been widely used in radios such as radar and electronic communication. The antenna array pattern synthesis, as a key problem to antenna arrays, has attracted much attention. The main task of the antenna array pattern synthesis is to adjust the excitation amplitudes, the phases, and the positions of array elements of the antenna array to obtain the pattern with required characteristics.

In the early study, due to the limited computing resource, some classic analytical techniques such as the Dolph–Chebyshev method and Taylor method [2] are used to optimize the excitation amplitudes of the array so as to suppress the sidelobe. Since the end of the 20th century, with the rapid development of computer technology, more and more random optimization algorithms have been applied to the antenna design. These highly flexible algorithms have few restrictions on the optimization objectives, which make them suitable to solve complicated and nonlinear optimization problems, and hence they show a better optimization performance and a higher degree of freedom in antenna array pattern synthesis. These algorithms have been used in various pattern synthesis problems, and with the help of them, optimization objectives such as sidelobe suppression, null depth and beamforming have been achieved. For example, the genetic algorithm (GA) [3] has been applied to optimize the excitations of uniform array elements [4]. Additionally, the particle swarm optimization (PSO) algorithm [5] and the enhanced flower pollination (EFPA) algorithm have been applied to optimize the distances between the array elements of the non-uniform arrays [6,7], and the differential evolution (DE) algorithm [8] has been used for the pattern synthesis of the time-modulated array [9]. However, the random optimization algorithms cannot always meet the design requirements of antenna arrays due to their low convergence rate, long running time, and/or being trapped in the local optimum. Therefore, there are still incentives to find a more efficient and practical random optimization algorithm.

The DE algorithm is prominent for the pattern synthesis of antenna array which utilizes the array element excitations as optimization variables. However, there are difficulties in the control parameter settings and the mutation strategy selection. Therefore, various algorithms are proposed such as the dynamic differential evolution (DDE) [10], the composite differential evolution (CoDE) [11], and the hybrid differential evolution algorithms [12]. Most of them, as well as some pertinently modified differential evolution algorithms [13,14], have been applied to the pattern synthesis of antenna arrays [15,16,17] and prove to enhance the optimization performance effectively. Among them, the JADE algorithm [18], a state-of-the-art improved DE algorithm, has been widely applied and shown strong performance in optimization problems because of its ability in the parameter adaptive adjustment. Besides, to solve the stagnation problem of DE algorithm, Shu-Mei Guo and Chin-Chang Yang proposed the successful-parent-selecting (SPS) framework [19] in 2015 that uses the success history in the iterations to select the parent vectors, which makes it easier for the DE algorithm to find the global optimum. 

Under the constraint of the beamwidth, the excitation amplitudes optimization of the linear antenna arrays with sidelobe suppression and null depth is considered in this paper. Several similar works have been completed with different algorithms [20,21,22,23], and they all show satisfactory results. However, in the above-mentioned works, the number of the array elements used in the simulations is small, and thus the power of the algorithms hasn’t been examined in the large-scale antenna arrays. Another less-noticed requirement is to shorten the optimal solution searching time so that the antenna arrays can respond to the changing situations more easily and quickly. In this paper, an SPS-JADE algorithm for antenna array pattern syntheses is designed by combining the aforementioned JADE algorithm with the SPS framework. This algorithm can find the optimal solution with fewer iteration numbers, and it is verified in the pattern synthesis simulations for the antenna arrays with more elements. In comparison with other random optimization algorithms, the SPS-JADE algorithm gives better optimization result in the pattern syntheses and has the potential to meet the requirements stated above with its excellent global optimization and rapid convergence feature.

The rest of this paper is organized as follows. In Section 2, the problem of linear antenna array pattern synthesis and its optimization model is introduced. In Section 3, the basic ideas and implementation steps of the classic DE algorithm, the JADE algorithm, and the SPS-JADE algorithm with SPS framework are introduced. In Section 4, the SPS-JADE algorithm is utilized for the simulations of pattern syntheses. Numerical results compared with other algorithms are presented and analyzed. Conclusions are given in Section 5.

## 2. Pattern Synthesis of Linear Antenna Array

**Figure 1:**
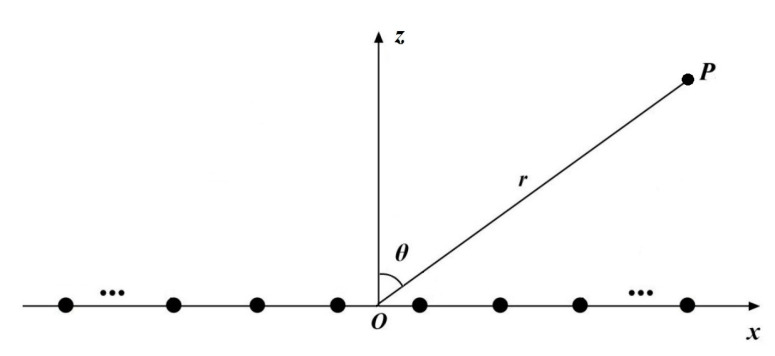
The geometry of the linear antenna array.

The far-field pattern is mainly related to the array factor when a linear antenna array consists of elements placed along the *x*-axis as shown in Figure 1. 

The array factor of a broadside linear array on the *x-z* plane is expressed as follows [24]:

(1) $$F_{A}(\theta ,x,I,\varphi )=\sum_{n=1}^{N}I_{n}e^{j(\frac{2\pi }{\lambda }x_{n}\sin\theta +\varphi_{n})},$$

where the elements of the vectors $x={\left[x_{1},x_{2},\ldots ,x_{N}\right]}^{T}$, $I={\left[I_{1},I_{2},\ldots ,I_{N}\right]}^{T}$, and $\varphi ={\left[\varphi_{1},\varphi_{2},\ldots ,\varphi_{N}\right]}^{T}$ are the position coordinates, the excitation amplitudes and phases of array elements, respectively. λ is the wavelength, θ is the steering angle of the antenna from the positive *z*-axis, and *N* is the number of array elements.

In this paper, we consider an *N*-element equally spaced linear symmetric antenna array with an adjacent element separation of λ/2. Consider the case that *N* is even, with the phases of all array elements zeroed in advance, Equation (1) becomes

(2) $$F_{A}(\theta ,x,I,\varphi )=2\sum_{n=1}^{N/2}I_{n}\cos[\pi (n-\frac{1}{2})\sin\theta ].$$

The excitation amplitudes are taken as the optimization variables and constrained within [0,1]. The sidelobe suppression, along with the beamwidth constraint, serves as the optimization objective. The objective function is given by

(3) $$f(I)=\frac{\underset{\theta \in S}{\max}(\left|F\right|)}{\max(\left|F\right|)}+\varepsilon \cdot \max\left\{0,FNBW-{FNBW}_{D}\right\},$$

where *S* is the area outside of the main beam in the pattern. The first term on the right side of the equation is the normalized maximum sidelobe level (MSL). FNBW denotes the first null beamwidth (FNBW), which is calculated as the angle difference between the minimum amplitude points nearest to the peak of the main beam on the left and right in the pattern. ${FNBW}_{D}$ is the desired FNBW. ε is the penalty factor set to 10^4^. For the anti-interference function, by adding a null depth term of which the penalty factor is set to 1, the objective function becomes

(4) $$f(I)=\;\frac{\underset{\theta \in S}{\max}(\left|F\right|)}{\max(\left|F\right|)}+\varepsilon \cdot \max\left\{0,\mathrm{FNBW}-{\mathrm{FNBW}}_{D}\right\}+\sum_{m=1}^{M}\frac{\left|F(\theta_{m}^{null})\right|}{\max(\left|F\right|)},$$

where $\theta_{m}^{null}$ is the given angle direction of the *m*th null. The optimization model can then be expressed as

(5) $$\left\{ \begin{array}{l} \mathrm{find}\;I=[I_{1},I_{2},\ldots ,I_{N/2}]\\ \min\{f(I)\}\\ s.t.\;\;0\leq I_{n}\leq 1,\;n\in \left\{1,2,\ldots ,N/2\right\} \end{array}\right.,$$

and the result of pattern synthesis can be obtained by optimizing this model with the given random optimization algorithm.

## 3. SPS-JADE Algorithm

### 3.1. Classic DE Algorithm

The simplicity, high efficiency and robustness of the classic DE algorithm proposed by Rainer Storn and Kenneth Price make it suitable for solving many nonlinear problems. Firstly, an initial population

(6) $$X_{0}=\{x_{i,0}{|x_{i,0}=[x_{1,i,0},x_{2,i,0},\ldots ,x_{D,i,0}]}^{T},i=1,2,\ldots ,NP\},$$

is randomly generated in the constrained optimization space, where *D* is the dimension of the variable, and *NP* is the population size. After initialization, the steps to update the population in each iteration can be divided into three operations: mutation, crossover, and selection.

The mutation operation is the process of generating the mutation vectors through a linear calculation of the parent vectors and the differential vectors. The expressions of the two most common strategies are given as follows:

1. “DE/rand/1”

(7) $$v_{i,G}=x_{r_{1},G}+F\cdot \left(x_{r_{2},G}-x_{r_{3},G}\right),$$

2. “DE/best/1”

(8) $$v_{i,G}=x_{best,G}+F\cdot \left(x_{r_{1},G}-x_{r_{2},G}\right),$$

where the subscripts $r_{1}$, $r_{2}$, and $r_{3}$ are three different integers chosen from {1,2,…,NP} randomly and not equal to *i*. $x_{best,G}$ is the optimal vector in the *G*th generation population, and *F* is the scaling factor, which is a constant within [0,1]. 

The crossover operation is the process of generating trial vectors $u_{i,G}$ by the binomial crossover between the mutation vectors and the parent vectors expressed as follows:

(9) $$u_{j,i,G}=\left\{ \begin{array}{l} v_{j,i,G},\;\mathrm{if}\;\;rand<CR\;\mathrm{or}\;j=j_{rand}\\ x_{j,i,G},\;\;\;\mathrm{otherwise}\;\;\;\;\;\;\;\;\;\;\;\;\;\;\;\;\;\;\;\;\;\;\;\;\;\;\;\;\;\;\;\;\;\; \end{array}\right.,$$

where CR∈[0,1] is the crossover rate. For each *i* and *j*, *rand* is a uniformly distributed number within [0,1]. $j_{rand}$ is an integer randomly chosen from [1, *D*] for each *i*, which guarantees the diversity of searching. The trial vectors out of the boundary constraints can be adjusted by

(10) $$u_{j,i,G}=\left\{ \begin{array}{l} (x_{j,i,G}+x_{j}^{low})/2,\;\;\;\;\mathrm{if}\;x_{j,i,G}<x_{j}^{low}\\ (x_{j,i,G}+x_{j}^{up})/2,\;\;\;\;\mathrm{if}\;x_{j,i,G}>x_{j}^{up}\; \end{array}\right.,$$

where $x_{j}^{low}$ and $x_{j}^{up}$ are the lower and upper boundary of the optimization space, which are set to 0 and 1, respectively as in Section 2.

Finally, the selection operation, which is greedy, is to choose the better individuals from the trial vectors and the parent vectors, and place them into the parent population of the next generation. As shown in Section 2, the purpose of the pattern synthesis is to find the minimum of the objective function. Hence, the specific operation can be expressed as

(11) $$x_{i,G+1}=\left\{ \begin{array}{l} u_{i,G},\;\mathrm{if}\;f(u_{i,G})<f(x_{i,G})\\ x_{i,G},\;\mathrm{otherwise}\;\;\;\;\;\;\;\;\;\;\;\;\;\;\;\;\;\;\;\;\;\;\;\; \end{array}\right.,$$

where f(⋅) is the objective function value. The selection operation is a successful update when satisfies $f(u_{i,G})<f(x_{i,G})$, and such condition is of great significance in the JADE algorithm and SPS framework introduced next.

### 3.2. JADE Algorithm

The JADE algorithm proposed by Jingqiao Zhang and Arthur C. Sanderson is an adaptive DE algorithm with an optional external archive that improves the performance of the classic DE algorithm. The improvements mainly consist of the selection of mutation strategies and the adaptive adjustment of control parameters.

The selection of mutation strategies seriously affects the balance between the search ability and the convergence rate. Therefore, it is significant to choose an appropriate mutation strategy. The JADE algorithm provides a compromise strategy by giving consideration to both sides, which is named as DE/current-to-*p*best/1 and can be expressed as

(12) $$v_{i,G}=x_{i,G}+F_{i,G}\cdot \left(x_{best,G}^{p}-x_{i,G}\right)+F_{i,G}\cdot \left(x_{r_{1},G}-x_{r_{2},G}^{A}\right),$$

where the subscripts $r_{1}$, $r_{2}$, are randomly chosen, and $r_{1}\neq r_{2}\neq i$. $x_{best,G}^{p}$ is a vector randomly selected from the top *p* × 100% of the current parent population sorted from best to worst, and *p* is a given parameter within [0,1]. $x_{r_{2},G}^{A}$ is a vector randomly selected from the union of the current parent population and the archive defined by the set of archived inferior solutions. The archive is one of the improvements of the JADE algorithm. It is initialized as empty with a maximum population size of *NP*. Whenever a successful update is completed, the replaced vector enters the archive. When the number of vectors in the archive is equal to *NP*, the newly entered one will randomly replace an original one. This procedure can expand the selection space of the difference vector and further enhance the search diversity.

It can be seen in Equation (12) that the scaling factor *F* is no longer a constant; instead, it is independently generated for each individual in the population at each generation. Similarly, the crossover rate *CR* is also a variable. These two parameters are generated by

(13) $$F_{i,G}=randc_{i}\left(\mu_{F,G},0.1\right),$$

(14) $$CR_{i,G}=randn_{i}\left(\mu_{CR,G},0.1\right),$$

and truncated to [0,1] (especially, $F_{i,G}$ will be regenerated if $F_{i,G}\leq 0$), where for each *i*, $randc_{i}(a,b)$ and $randn_{i}(a,b)$ are random numbers generated by a Cauchy distribution that serves to diversify the scaling factors, and a Gaussian distribution, respectively, with location parameter *a* and scale parameter *b*. $\mu_{F,G}$ and $\mu_{CR,G}$ are initialized as given parameters $\mu_{F,0}$ and $\mu_{CR,0}$, respectively, and updated after the selection operation at each generation:

(15) $$\mu_{F,G+1}=(1-c)\cdot \mu_{F,G}+c\cdot \frac{\sum_{F_{i,G}\in S_{F}}F_{i,G}^{2}}{\sum_{F_{i,G}\in S_{F}}F_{i,G}},$$

(16) $$\mu_{CR,G+1}=(1-c)\cdot \mu_{CR,G}+c\cdot \mathrm{mean}(S_{CR}),$$

where mean(⋅) denotes the numerical average, *c* is a given constant within [0,1]. $S_{F}$ and $S_{CR}$ are the sets of scaling factors and crossover rates corresponding to all individuals that have completed the successful updates in the current population, which guide the selection of control parameters at the next generation and realize the adaptive adjustment to overcome the lack of adaptability to the optimization problems. Therefore, this improvement is helpful to adjust the mutation and crossover operation timely and to further solve the pattern synthesis problems pertinently with a high convergence rate to shorten the searching time.

### 3.3. SPS-JADE Algorithm with SPS Framework

Although the JADE algorithm can improve the convergence performance, problems may arise in the multiple-dimensional cases such as the pattern syntheses. When the optimization dimension increases, the aggregate of the solutions will grow exponentially, resulting in a sharp increase in the number of local optima, and the high convergence rate will lead to the global searching trapping in these local optima. Consequently, it will be harder and take longer to find the new optimal solutions in the population when the DE algorithm is applied to the large-scale antenna arrays pattern syntheses with a large number of the array elements. This phenomenon is called stagnation, and to overcome this, here, the SPS framework that can provide a timely response to the stagnation occurring is utilized to further improve the optimization ability of the DE algorithm and the efficiency of the pattern syntheses.

The core of the SPS framework is to use different parent vectors in the mutation and crossover operation. In the DE algorithm, when the stagnation occurs, a population individual cannot be successfully updated for a long time. In this case, the last *NP* vectors that complete the successfully updates in the history, dubbed the successful parents, will be chosen as the parent vectors instead of the vectors selected at the previous generation. The algorithm can then be guided out of the stagnation by successful parents with a higher potential of searching. The standard to measure whether stagnation occurs is the total number of selection operations performed for each population individual over the duration that the individual continuously fails to be updated. When this number is greater than the given stagnation tolerance *Q*, the stagnation occurs, and the algorithm will use the successful parents to update the population. This novel method of the parent selection makes the algorithm keep exploring a better solution efficiently without the reduction of the convergence rate, which shows its potential to solve the high dimensional optimization problems. 

**Figure 2:**
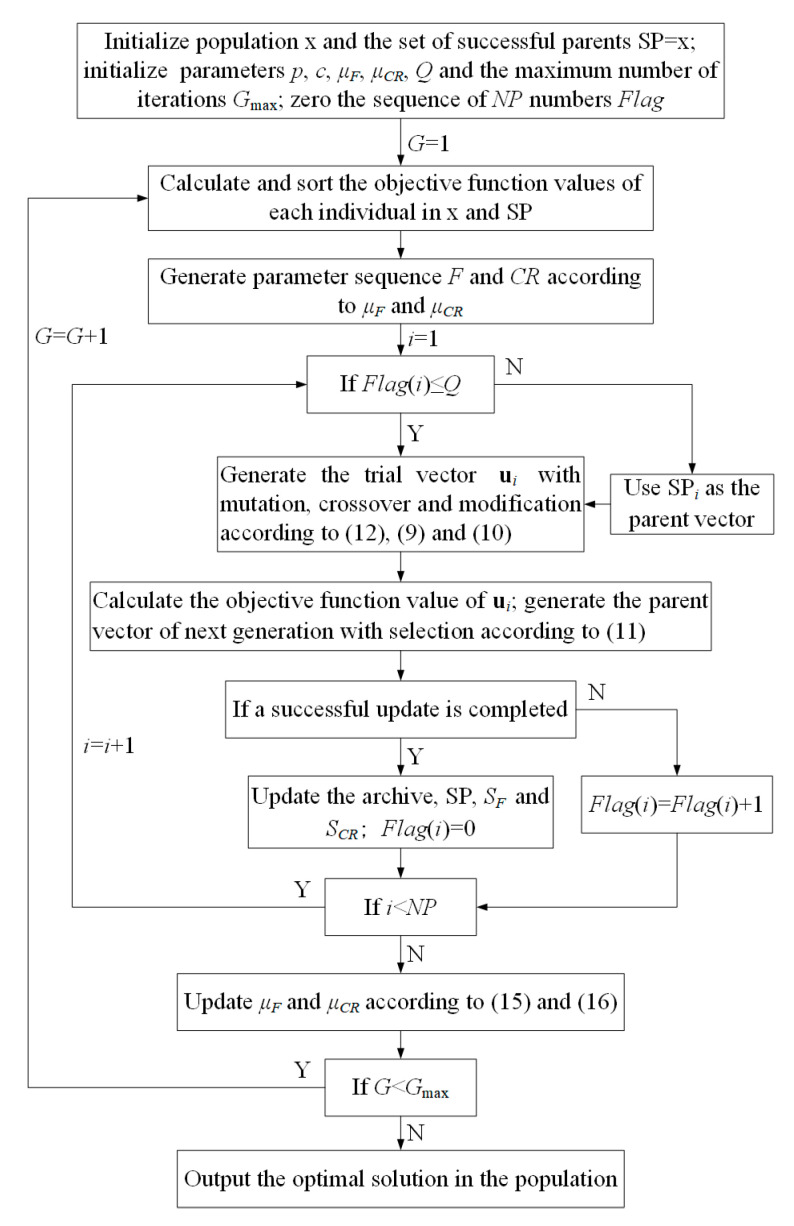
Flowchart of the SPS-JADE algorithm.

Theoretically, the SPS framework can be applied to the general DE algorithms. In this paper, the introduced JADE algorithm is combined with the SPS framework as the SPS-JADE algorithm, which is further applied to the pattern synthesis of the linear antenna arrays introduced in Section 2. The flowchart of the SPS-JADE algorithm is shown in Figure 2.

## 4. Numerical Analysis and Results

### 4.1. Parameter Settings 

The linear symmetric antenna array used here has 40 array elements, which is relatively more than the examples in the previous works mentioned in Section 1 [20,21,22,23], with an adjacent element spacing of λ/2. In the following simulation experiments, the parameters are chosen by referring to [18,19] and trained by using simulations under the optimization model of the antenna array to achieve a better optimization effect. For the SPS-JADE algorithm, p=0.05, c=0.1, $\mu_{F,0}=0.7$, $\mu_{CR,0}=0.8$, and Q=10. Comparisons are made with other random optimization algorithms by using the same example and appropriate parameters setting. For the classic DE algorithm with DE/best/1, F=0.7 and CR=0.8; for the improved GA algorithm in [25], the mutation probability $p_{m}=0.3$; for the jDE algorithm [26], the initial control parameters $F_{0}=0.5$, $CR_{0}=0.5$; for the SaDE algorithm [27], the learning period LP=10; for the DEGL algorithm [28], the mutation parameters α=β=F=0.7, the crossover rate CR=0.8, and the neighborhood size is the 10% of the population size; and for the JADE algorithm without the SPS framework, p=0.05, c=0.1, $\mu_{F,0}=0.7$, and $\mu_{CR,0}=0.8$. In all these algorithms, the desired FNBW $FNBW_{D}=10{}^{\circ}$. The angle resolution is set to 0.02°. The population size and the maximum number of the iterations are set to 50 and 300, respectively, to examine the convergence performance of the algorithms.

### 4.2. Simulation Experiments Results

#### 4.2.1. Experiment A

Each algorithm runs 30 times independently to simulate the 40-element linear symmetric antenna array synthesis with sidelobe suppression, in which Equation (4) is used as the objective function. 

**Figure 3:**
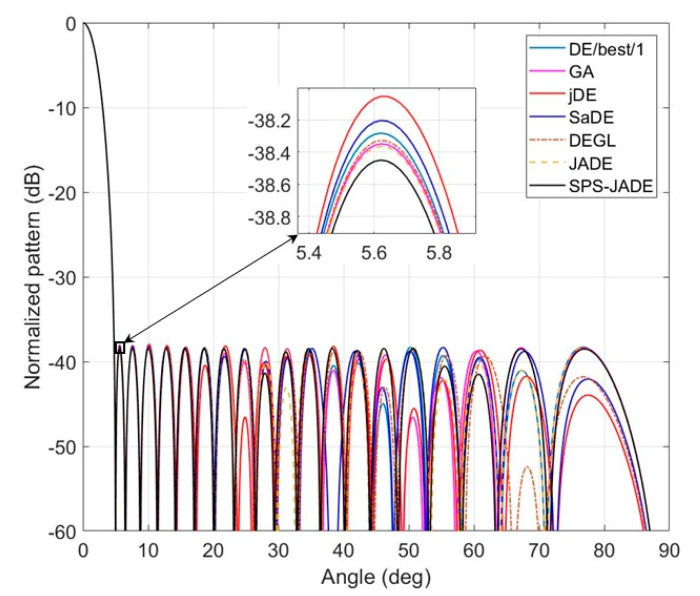
The optimal patterns for the linear antenna array.

**Figure 4:**
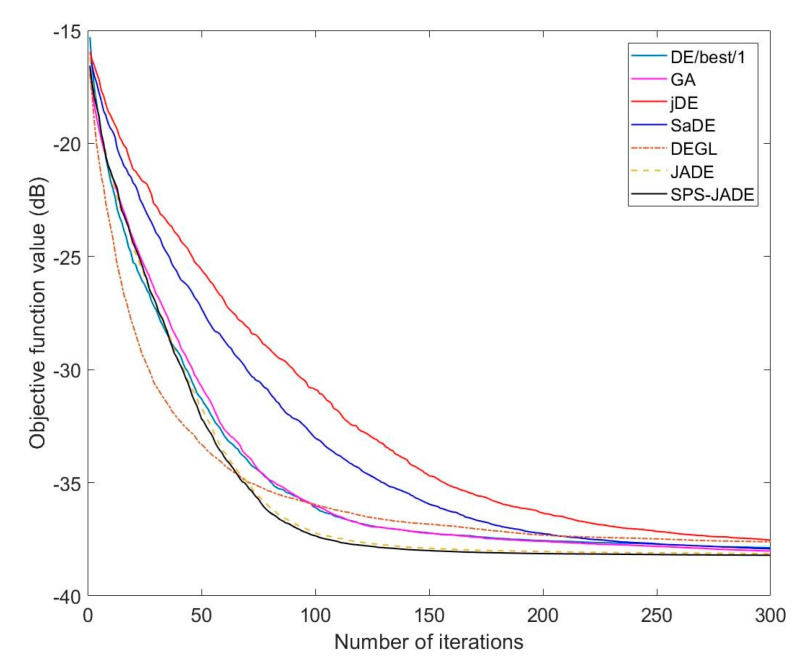
Average convergence rates with sidelobe suppression.

The synthesis results of the normalized MSLs obtained by different algorithms are given in Table 1. The optimal excitation amplitudes of the array elements computed by SPS-JADE algorithm are given in Table 2 with the element number that increases along the positive direction of the *x*-axis in Figure 1. Figure 3 shows the optimal patterns optimized by different algorithms. Since the antenna pattern is symmetrical along the steering angle θ=0°, only a part of the antenna pattern with θ∈[0°,90°] is shown here. It can be seen that the sidelobe suppression results of the SPS-JADE algorithm are generally better than the other algorithms. The normalized MSL obtained by the SPS-JADE algorithm is around −38.45 dB, which validates its superiority in the global optimum searching. In addition, from Table 1, we can see that the SPS-JADE algorithm performs more stably and robustly during the multiple independent runs with a standard deviation of 0.1468 dB, which is lower than other algorithms. The average convergence rates of different algorithms are shown in Figure 4. We can see that the SPS-JADE algorithm converges within 150 iterations, which shows its outstanding convergence performance. It is clear to see the superiority of the SPS-JADE algorithm in the convergence rate, compared with the classic DE algorithm with DE/best/1, the improved GA algorithm, the jDE algorithm, and the SaDE algorithm. Despite that the convergence rate of the DEGL algorithm is faster in the early iterations, it is generally inferior to the SPS-JADE algorithm. Compared with the JADE algorithm, the convergence rate of the SPS-JADE algorithm is only slightly improved by using the SPS framework.

In order to further study the optimization performance of algorithms in the pattern synthesis, we select the SPS-JADE algorithm and the JADE algorithm with the best two performance in the experiment for the pattern synthesis efficiency test and the results in the above experiment will also be used for the analysis. In this paper, the pattern synthesis efficiency is examined by the total number of evaluations required for the objective function to attain the given ‘value to reach’ (VTR) [16]. Since the population size and the maximum number of iterations are 50 and 300, respectively, the maximum number of objective function evaluations is 15,000 for each run. During this period, the number of the independent runs that successfully reach the VTR is represented as $N_{S}$, and the success rate $r_{S}=N_{S}/30$. Four target values with high success rates and certain reference value are given as the VTRs. The pattern synthesis efficiency test results are shown in Table 3, where ${\mathrm{FE}}_{\min}$, ${\mathrm{FE}}_{\max}$, and ${\mathrm{FE}}_{\mathrm{avg}}$ are the minimum, the maximum and the average number of the objective function evaluations taken to reach the VTRs in 30 runs, respectively. From the table, to achieve four different VTRs, the ${\mathrm{FE}}_{\mathrm{avg}}s$ with the SPS-JADE algorithm are less than that with the JADE algorithm, which means that the SPS-JADE algorithm tends to achieve the optimization objectives before the JADE algorithm. The success rates of the SPS-JADE are also slightly higher than the JADE. In conclusion, these results intuitively show that the SPS-JADE algorithm has higher optimization efficiency and better convergence performance in the pattern synthesis, which demonstrates the effectiveness of our algorithm with the improvement induced by using the SPS framework.

#### 4.2.2. Experiment B

Assume the null at the direction of θ=24°. The other parameter settings are the same as in Experiment A. Seven algorithms above are used to in the simulation experiment of the pattern synthesis of the same 40-element linear symmetric antenna array to simultaneously achieve null depth and sidelobe suppression. Thirty independent runs are repeated for each algorithm, where Equation (5) is used as the objective function.

**Figure 5:**
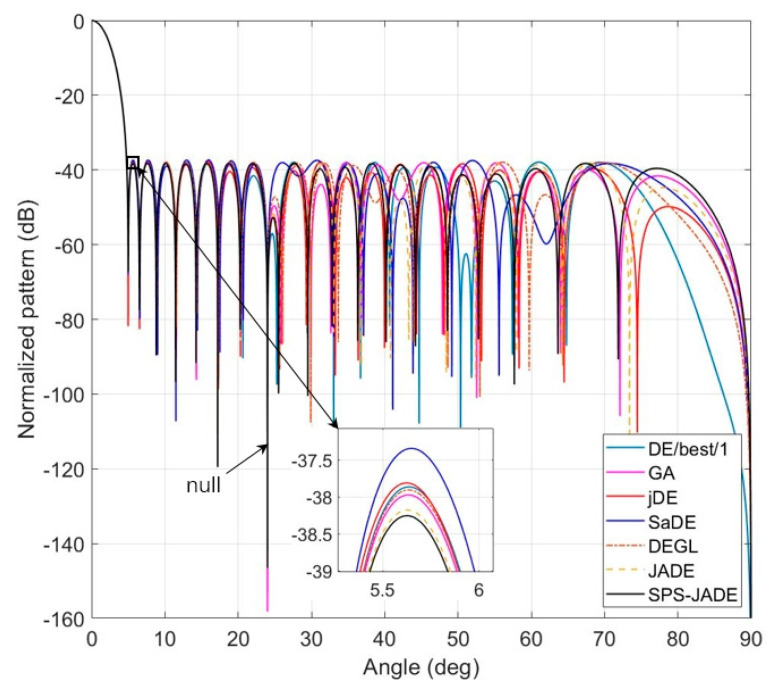
The optimal patterns with the null at *θ* = 24° for the linear antenna array.

**Figure 6:**
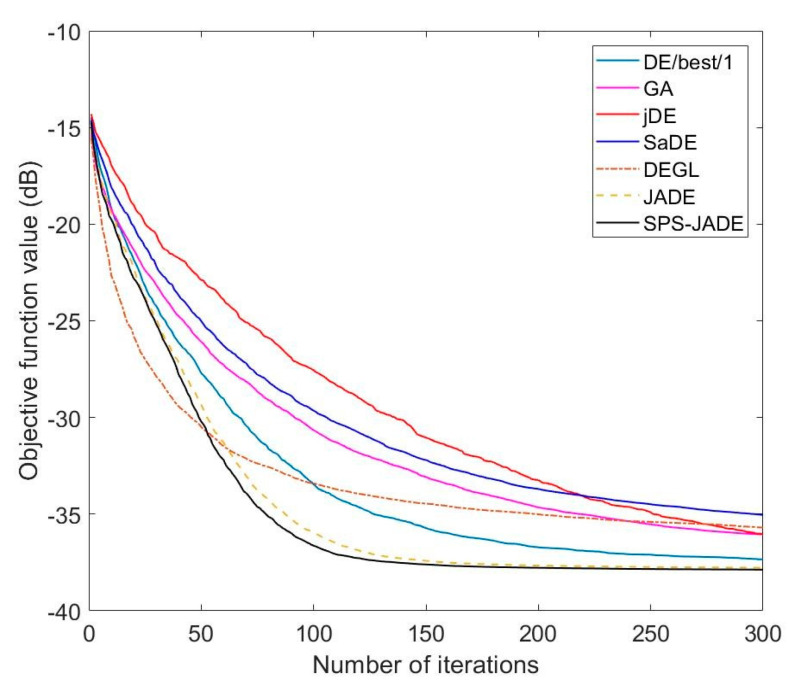
Average convergence rates with sidelobe suppression and null depth.

The best, the worst, the average values, and the standard deviations of the normalized MSLs by different algorithms are given in Table 4. The best, the worst, and the average values of the normalized null depths are given in Table 5, and the optimal patterns are shown in Figure 5. Table 6 presents the optimal excitation amplitudes of the array elements computed by the SPS-JADE algorithm. It can be seen that the SPS-JADE algorithm, which obtains the normalized MSL below −38.25 dB, is still superior to the other algorithms in the sidelobe suppression despite considering the successfully generating deep null with the null depth lower than −130 dB at the desired direction. Furthermore, it is shown that the SPS-JADE algorithm can remain robust with a low standard deviation of the normalized MSLs about 0.17 dB. The average convergence rates of different algorithms are shown in Figure 6. Comparing with Figure 4, we can see that the convergence performance of each algorithm is similar to that in Experiment A, and the SPS-JADE algorithm again shows its advantage in the convergence rate.

## 5. Conclusions

In this paper, a SPS-JADE algorithm for the antenna array pattern synthesis is designed. It improves the shortcomings of the classical DE algorithm by making the control parameters adaptive and by giving a better mutation strategy. In particular, the SPS framework used in the algorithm solves the stagnation problem which occurs in various DE algorithms. The SPS-JADE algorithm is applied to the pattern synthesis of a 40-element linear symmetric antenna array with the sidelobe suppression and null depth under the beamwidth constraint of optimizing the excitation amplitudes of array elements. By using the SPS-JADE algorithm, the normalized MSL of the antenna array can be reduced to around −38.45 dB, and this value is around −38.25 dB with the null depth lower than −130 dB, which are both the lowest among the algorithms discussed in this paper. The small average and standard deviations of MSLs mean that the SPS-JADE algorithm can stably obtain satisfactory results. Furthermore, the pattern synthesis efficiency of the SPS-JADE algorithm is compared with the JADE algorithm to validate the effect of the SPS framework in speeding up the global optimum searching. In conclusion, these simulation results show that, for the antenna array pattern synthesis, the SPS-JADE algorithm has better performance in terms of the global search ability, the convergence rate, and robustness, which demonstrates its great potential for the design of large-scale antenna arrays.

## Figures and Tables

**Table 1:** Synthesis results of the normalized MSLs for the linear antenna array with sidelobe suppression.

Algorithm	Best (dB)	Worst (dB)	Average (dB)	Std. (dB)
DE/best/1	−38.2821	−37.3611	−37.8710	0.2084
GA	−38.3459	−37.6051	−38.0196	0.1887
jDE	−37.9625	−36.7904	−37.5397	0.2577
SaDE	−38.2037	−37.4546	−37.9220	0.1622
DEGL	−38.3228	−36.1336	−37.6120	0.5901
JADE	−38.3639	−37.4839	−38.1391	0.1811
SPS-JADE	−38.4496	−37.7394	−38.2081	0.1468

**Table 2:** The optimal excitation amplitudes of array elements computed by SPS-JADE algorithm with sidelobe suppression.

Element Number	Excitation Amplitude	Element Number	Excitation Amplitude
1, 40	0.1853	11, 30	0.6274
2, 39	0.1326	12, 29	0.6941
3, 38	0.1679	13, 28	0.7436
4, 37	0.2156	14, 27	0.8028
5, 36	0.2614	15, 26	0.8496
6, 35	0.3085	16, 25	0.9015
7, 34	0.3830	17, 24	0.9325
8, 33	0.4332	18, 23	0.9580
9, 32	0.5012	19, 22	0.9771
10, 31	0.5561	20, 21	0.9916

**Table 3:** Results of the pattern synthesis efficiency test with sidelobe suppression.

VTR (dB)	SPS-JADE				JADE			
	$r_{S}$	$FE_{\min}$	$FE_{\max}$	$FE_{avg}$	$r_{S}$	$FE_{\min}$	$FE_{\max}$	$FE_{avg}$
−37.4	100%	4030	7065	5060	100%	4242	14,332	5470
−37.6	100%	4583	8535	5563	96.7%	4504	9530	5742
−37.8	96.7%	4865	9952	6132	96.7%	5001	13,147	6773
−38.0	90%	5552	12,236	7252	83.3%	5754	11,430	7810

**Table 4:** Synthesis results of the normalized MSLs for the linear antenna array with sidelobe suppression and null depth.

Algorithm	Best (dB)	Worst (dB)	Average (dB)	Std. (dB)
DE/best/1	−37.8559	−35.9365	−37.3418	0.3833
GA	−37.9721	−30.5613	−36.0488	1.7067
jDE	−37.6730	−33.7738	−36.0254	0.9156
SaDE	−37.3390	−31.9234	−35.0410	1.2500
DEGL	−37.8964	−29.9828	−35.6964	2.1167
JADE	−38.1666	−37.3229	−37.7729	0.2104
SPS-JADE	−38.2521	−37.3910	−37.8737	0.1703

**Table 5:** Synthesis results of the normalized null depths for the linear antenna array with sidelobe suppression and null depth.

Algorithm	Best (dB)	Worst (dB)	Average (dB)
DE/best/1	−130.4027	−74.6508	−92.1970
GA	−167.9265	−103.7220	−118.4906
jDE	−103.5643	−61.3954	−73.6811
SaDE	−112.0015	−73.8613	−98.3941
DEGL	−142.5442	−77.2106	−106.6899
JADE	−166.6318	−111.3316	−131.7949
SPS-JADE	−162.1386	−105.3397	−130.8599

**Table 6:** The optimal excitation amplitudes of array elements computed by SPS-JADE algorithm with sidelobe suppression and null depth.

Element Number	Excitation Amplitude	Element Number	Excitation Amplitude
1, 40	0.1736	11, 30	0.6040
2, 39	0.1271	12, 29	0.6776
3, 38	0.1710	13, 28	0.7447
4, 37	0.2173	14, 27	0.8021
5, 36	0.2607	15, 26	0.8358
6, 35	0.2948	16, 25	0.8740
7, 34	0.3699	17, 24	0.9205
8, 33	0.4366	18, 23	0.9613
9, 32	0.4954	19, 22	0.9698
10, 31	0.5573	20, 21	0.9682
